# Is Serum Zinc Associated with Pancreatic Beta Cell Function and Insulin Sensitivity in Pre-Diabetic and Normal Individuals? Findings from the Hunter Community Study

**DOI:** 10.1371/journal.pone.0083944

**Published:** 2014-01-08

**Authors:** Khanrin P. Vashum, Mark McEvoy, Abul Hasnat Milton, Md. Rafiqul Islam, Stephen Hancock, John Attia

**Affiliations:** 1 Centre for Clinical Epidemiology and Biostatistics (CCEB), School of Medicine and Public Health, The University of Newcastle, and Hunter Medical Research Institute, Newcastle, New South Wales, Australia; 2 Ministry of Health and Family Welfare, Government of Bangladesh, Dhaka, Bangladesh; Brigham & Women's Hospital, and Harvard Medical School, United States of America

## Abstract

**Aim:**

To determine if there is a difference in serum zinc concentration between normoglycaemic, pre-diabetic and type-2 diabetic groups and if this is associated with pancreatic beta cell function and insulin sensitivity in the former 2 groups.

**Method:**

Cross sectional study of a random sample of older community-dwelling men and women in Newcastle, New South Wales, Australia. Beta cell function, insulin sensitivity and insulin resistance were calculated for normoglycaemic and prediabetes participants using the Homeostasis Model Assessment (HOMA-2) calculator.

**Result:**

A total of 452 participants were recruited for this study. Approximately 33% (N = 149) had diabetes, 33% (N = 151) had prediabetes and 34% (N = 152) were normoglycaemic. Homeostasis Model Assessment (HOMA) parameters were found to be significantly different between normoglycaemic and prediabetes groups (p<0.001). In adjusted linear regression, higher serum zinc concentration was associated with increased insulin sensitivity (p = 0.01) in the prediabetic group. There was also a significant association between smoking and worse insulin sensitivity.

**Conclusion:**

Higher serum zinc concentration is associated with increased insulin sensitivity. Longitudinal studies are required to determine if low serum zinc concentration plays a role in progression from pre-diabetes to diabetes.

## Introduction

Diabetes, a disorder of metabolism with defects in either insulin secretion, insulin action or both, is increasing globally due to population growth, aging, urbanization, unhealthy eating habits, and increasing prevalence of obesity and physical inactivity [1]. Diabetes is a leading cause of morbidity and mortality with an estimated 346 million adults being affected worldwide in 2011 [1]. The prevalence is continuing to rise and is expected to double between 2005–2030. [1], [2].

Type 2 diabetes is often asymptomatic and may remain undiagnosed for several years [3]. It is characterized by insulin resistance, hyperinsulinaemia, beta cell dysfunction and subsequent beta cell failure [4]. Numerous influences on the onset of diabetes have been proposed, one of which may be the abnormal homeostasis of trace elements such as zinc [5]. [6]. Zinc is involved in the synthesis, storage, and secretion of monomeric insulin, as well as conversion to a dimeric form for storage and secretion as crystalline insulin [7], [8]. Zinc is essential in insulin action and carbohydrate metabolism [9]. Oxidative stress also plays an important role in the pathogenesis of diabetes and its complications, and zinc is a structural component of key antioxidant enzymes such as superoxide dismutase, which is vital for intra- and extracellular antioxidant defence [10].

Animal models have shown that peripheral insulin resistance contributes to impaired glucose tolerance in non-diabetic, zinc deficient rats [11]. Oral administration of zinc complex in KKA(y) mice daily also caused significant improvements in hyperglycaemia, glucose intolerance and insulin resistance [12]. Conversely, a zinc deficient diet led to increased fasting blood glucose levels and reduced circulating insulin in db/db mice [13]. Human studies on the other hand have had conflicting results regarding zinc supplementation in type 2 diabetes. A lower incidence of type 2 diabetes has been reported in women who had a higher intake of dietary zinc [14]. A Cochrane review found that there was insufficient evidence to suggest the use of zinc supplementation in the prevention of type 2 diabetes [15]. By contrast, a systematic review and meta-analysis of 25 articles, which included 22 studies on type 2 diabetes, concluded that zinc supplementation has beneficial effects on glycaemic control [16]. The review however had several limitations including differences in zinc doses, sample size, study duration, limited availability of data on zinc intake and variation in baseline parameters.

Pre-diabetes is a condition that increases the risk of developing type 2 diabetes and often precedes diabetes. In Australia the prevalence of pre-diabetes is estimated at 16.4%, more than double the prevalence of type-2 diabetes [17]. Pre-diabetic individuals have a six fold increased risk of developing type 2 diabetes compared with those with normal glucose values [17]. People with Pre-diabetes have impaired fasting glucose (IFG), impaired glucose tolerance (IGT), or both; blood glucose is higher than normal but not high enough to be classified as diabetes and this state is reversible [17]. Reversion to normal glucose tolerance occurs in about 25% over 3–5 years of observation, while the rest remain stable or progress to overt diabetes. However, with longer observation, the majority of individuals with IFG or IGT appear to develop diabetes [18]. People in the pre-diabetic stage are not only at an increased risk of developing diabetes but also have an increased risk of developing cardiovascular and other macrovascular disease [17].

This study therefore set out to determine if there is a difference in serum zinc concentration between normal, pre-diabetic and type-2 diabetic groups of community-dwelling men and women aged 55–85 years. A further aim was to determine if serum zinc concentration is associated with pancreatic beta cell function and insulin sensitivity in pre-diabetic and normal individuals. The use of a pre-diabetic group who has not yet developed nephropathy is an important improvement in establishing that low zinc is not simply an epiphenomenon due to loss of zinc secondary to diabetic nephropathy.

## Methods

A random sample of participants aged between 55 and 85 years was selected from the Hunter Community Study (HCS), a cohort of community-dwelling men and women in Newcastle, New South Wales (NSW), Australia. Approval to conduct the research was granted by the University of Newcastle Human Research Ethics Committees and Hunter New England Research Ethics. This study has been described in detail elsewhere [19]. In brief, participants were randomly selected from the NSW State Electoral roll and contacted between mid December 2004 and May 2007. A modified Dillman recruiting strategy [20] was used whereby two letters of introduction and an invitation to participate were posted to the selected persons. An HCS research assistant telephoned persons who did not respond to initial postal contacts. If contact was not established after five attempts, the individual was classified as a non-responder. Persons who could not speak English and those living in a residential aged care facility were deemed ineligible. Once written consent to participate was obtained from eligible study subjects, they were asked to complete a series of self-reported postal questionnaires.

In addition to completing the postal questionnaires, participants were invited to attend the HCS data collection centre (clinic) to enable a series of clinical measures to be ascertained. The clinic measures included blood pressure, height, weight, waist-circumference and blood collection for routine biochemical analysis (including fasting blood glucose). The clinic measures assessed also included consent to link study information with data from Medicare Australia (Medicare and Pharmaceutical Benefit Scheme) and local health databases.

### Classification of diabetic status

As part of the clinical assessment in the HCS, fasting blood glucose was measured and participants were categorized into three groups according to American Diabetic Association (ADA) guidelines:

- Normal (normoglycemic): fasting blood glucose concentration <5.6 mmol/l,
- Prediabetic: fasting blood glucose concentration 5.6–6.9 mmol/l, and
- Diabetic: fasting blood glucose concentration ≥7 mmol/l or previously diagnosed as diabetic or currently taking diabetic medications (oral hypoglycaemic agents, insulin etc).

### Measurement of serum zinc and serum insulin

Blood was collected in EDTA tubes and centrifuged at 4°C and 3000 g for 10 min. to separate serum, which was stored for three years at −80°C before analysis. Serum zinc concentration was measured using Inductively Coupled Plasma Mass Spectrometry (ICP-MS, Perkin Elmer Sciex) as ICP-MS has greater precision and sensitivity compared to the older classic atomic absorption spectroscopy (AAS) technique often used to measure zinc. Analysis of serum insulin was performed using a BECKMAN-COULTER ‘DXI 800’.

### Calculation of HOMA parameters

Measured serum insulin and fasting blood glucose concentration was used to calculate steady state beta cell function (%B), insulin sensitivity (%S) and insulin resistance (IR) for normal and prediabetic participants by using the Homeostasis Model Assessment (HOMA2) calculator (University of Oxford, website; http://www.dtu.ox.ac.uk/homacalculator/index.php). The HOMA2 calculator is an updated version (computer model) of the original HOMA model (HOMA1). HOMA2 also accounts for variations in hepatic and peripheral glucose resistance and the reduction of peripheral glucose-stimulated glucose uptake. However, the HOMA2 calculator is not appropriate for use in frank diabetes as it needs further validation in this group [21].

### Measurement of other exposure variables

Participant's age, gender, education, income, smoking, alcohol intake, self-reported medical history (clinician diagnosis), self reported medications and use of zinc supplements or multivitamins with zinc, Body Mass Index (BMI) and Glomerular filtration rate (GFR) were collected. Education was categorized as primary school only, secondary school not completed, secondary school completed, trade or technical college qualification, and University or other tertiary qualification. Smoking was categorized as current, never, or former smoker. Alcohol intake was quantified and classified according to Australian National Health & Medical Research (NHMRC) guidelines [22] as number of drinks per month. BMI was calculated from participants' height and weight, which was measured during their clinic visit and grouped according to the guidelines for BMI classification by the World Health Organization (WHO).

### Statistical analysis

To detect a mean difference as low as 1.7 mmol/L (±5 mmol/L) in serum zinc concentration among three groups (normal, pre-diabetic and diabetic) with 95% confidence interval and 80% power, a sample size of 375 was calculated. Assuming a 15% refusal and dropout rate, recruiting a total of 450 eligible participants was estimated to be sufficient to observe the differences for this study, i.e. 150 in each group. The statistical analysis was performed using STATA software version 11.0 supplied by STATA Corporation, Texas, USA. Initially, baseline characteristics of the study participants compared by the participants' fasting blood glucose status and presented as mean, standard deviation and proportion were analysed using a chi-square test for categorical variables and analysis of variance (ANOVA) for continuous variables. For markers that did not follow a normal distribution, non-parametric tests were performed.

Homeostasis Model Assessment (HOMA2) calculator was used to calculate the HOMA parameters i.e. beta cell function (%B), insulin sensitivity (%S) and insulin resistance (IR) for normal and prediabetic participants. Multiple linear regression analyses were then performed for all three HOMA parameters for normoglycaemic and prediabetic group separately to determine factors associated with each outcome. Number of medications used was considered in the multivariate analyses, but dropped out of final models (except for insulin resistance in the pre-diabetic group).

## Results

A total of 452 participants with measurement of fasting blood glucose concentration were randomly recruited for this study. The baseline characteristics of the participants according to their fasting blood glucose concentration are shown in Table 1. Of the participants, roughly a third (N = 149) were diabetic, a third prediabetic (N = 151) and a third (N = 152) were normoglycaemic. Participants were roughly evenly split between males and females. Age was found to be significantly different with an overall average age of 66.6 years (±7.5), however the normoglycaemic group (65.6±7.3 years) was slightly younger compared to the diabetic group (67.9±7.7 years). Significant differences among the groups were also seen in household income, BMI, hypertension status, use of antihypertensive medication and the number of medications that the participants were taking. Overall, more than 80% of the participants across all the groups were on some form of medication. Only 6% (N = 27) of the total participants were taking zinc supplements or multivitamins with zinc but this was not significantly different between the groups. More than 50% in the normal group had never smoked but the rate of current smokers was similar across all the groups. Alcohol intake (number of drinks/month) was similar across the three groups with roughly 50% being classified as safe drinkers. The mean glomerular filtration rate (GFR) was also found to be similar across the three groups.

**Table 1:** Baseline characteristics by participants fasting blood glucose status.

Characteristic	Overall (n = 452)	Normal (n = 152)	Pre-diabetic (n = 151)	Diabetic (n = 159)	p-value
**Age (mean±SD)**	66.6 (7.4)	65.6 (7.3)	66.3 (7.0)	67.9 (7.7)	0.03
**Sex (n, %)**					>0.05
**Male**	238 (53.72)	69 (46.62)	89(60.54)	80 (54.05)	
**Female**	205 (46.28)	79(53.38)	58(39.46)	68(45.95)	
**Education (n, %)**					>0.05
**Primary schooling only**	14 (3.17)	7 (4.73)	1 (0.68)	6 (4.08)	
**Secondary schooling completed**	108 (24.43)	41 (27.70)	36 (24.49)	31 (21.09)	
**Secondary schooling not completed**	98 (22.17)	29 (19.59)	28 (19.05)	41(27.89)	
**Trade qualification or TAFE**	103 (23.30), 89 (20.14)	39 (6.35), 24 (16.22)	34 (23.13), 38 (25.85)	30(20.41), 27(18.37)	
**University or other tertiary study**	24 (5.43)	5 (3.38)	9 (6.12)	10 (6.80)	
**Others**	6 (1.36)	3 (2.03)	1 (0.68)	2 (1.36)	
**Household income/year (n,%)**					0.003
**Less than 5000**	8 (1.81)	1 (0.68)	2(1.36)	5 (3.40)	
**$5000–$9,999 per year**	22 (4.98)	7 (4.73)	8 (5.44)	7 (4.76)	
**$10,000–$19,999 per year**	98 (22.17)	25 (16.89)	31 (21.09)	42 (28.57)	
**$20,000–$29,999 per year**	92 (20.81)	31 (20.95)	26 (17.69)	35 (23.810	
**$30,000–$39,999 per year**	47 (10.63)	27 (18.240	11 (7.48)	9 (6.12)	
**$40,000–$49,999 per year**	38 (8.60)	13 (8.78)	15 (10.20)	10 (6.80)	
**$50,000–$69,999 per year**	51 (11.54)	22 (14.86)	17 (11.56)	12 (8.16)	
**$70 000 or more per year**	61 (13.80)	18 (12.160	30 (20.41)	13 (8.84)	
**Missing**	25(5.66)	4 (2.70)	7 (4.76)	14 (9.52)	
**Smoking status (n, %)**					>0.05
**Smoked never**	209 (47.18)	77 (52.03)	63 (42.86)	69 (46.62)	
**Previous smoker**	192 (43.34)	59 (39.86)	70 (47.62)	63 (42.57	
**Current smoker**	35 (7.90)	11 (7.43)	13 (8.84)	11 (7.43)	
**Missing**	7 (1.58)	1 (0.68)	1 (0.68)	5 (3.38)	
**Alcohol use (n, %)**					>0.05
**No**	91 (20.54)	33 (22.30)	19 (12.93)	39 (26.35)	
**Safe drinker**	236 (53.27)	83 (56.08)	81 (55.78)	71 (49.97)	
**Moderate drinker**	28 (6.32)	9 (6.08)	11 (7.48)	8 (5.41)	
**Hazardous drinker-binge**	27 (6.09)	8 (5.41)	11 (7.48)	8 (5.41)	
**Hazardous drinker-chronic**	18 (4.06)	5 (3.38)	9 (6.12)	4 (2.70)	
**Missing**	43 (9.71)	10 (6.76)	15 (10.20)	18 (12.16)	
**Hypertension (n, %)**					<0.001
**Yes**	237 (47.57)	61 (40.13)	80 (52.98)	96 (64.43)	
**No**	215 (52.34)	91 (59.87)	71 (47.02)	53 (35.57)	
**BMI (n, %)**					<0.001
**<25**	49 (10.89)	28 (18.54)	10 (6.67)	11 (7.38)	
**25.00–29.99**	185 (41.11)	75 (49.67)	63 (42)	47 (31.54)	
**≥30**	216 (48)	48 (31.79)	77 (51.33)	91 (61.07)	
**Taking any medicines (n, %)**					<0.001
**Yes**	387 (85.62)	122 (80.26)	126 (83.44)	139 (93.29)	
**No**	64 (14.38)	30 (19.74)	25 (16.56)	10 (6.71)	
**Taking antihypertensive (n, %)**					<0.001
**Yes**	243 (53.76)	60 (39.47)	80 (52.98)	103 (69.13)	
**No**	209 (46.24)	92 (60.53)	71 (47.02)	46 (30.87)	
**Taking anti diabetic (n, %)**					
**Yes**	78 (17.26)			78 (52.35)	
**No**	374 (82.74)	152 (100)	151 (100)	71 (47.65)	
**Taking zinc supplements or multivitamins with zinc (n, %)**					>0.05
**Yes**	27 (5.97)	9 (5.92)	6 (3.97)	12 (8.05)	
**No**	425 (94.03)	143 (94.08)	145 (96.03)	137 (91.95)	
**GFR (ml/mt) (mean± SD)**	75.3 (17)	77.4 (17.3)	73.9 (14.4)	74.5 (18.6)	>0.05

Table 2 shows the median of the blood/serum laboratory findings and HOMA parameters, as these biochemical and HOMA parameters were not normally distributed. Median fasting serum glucose concentration was 4.8 mmol/L, 5.8 mmol/L and 6.8 mmol/L in normalglycaemic, pre-diabetic and diabetic participants respectively. The median serum zinc concentration was found to be similar across all three groups (13 umol/L) however; median serum insulin concentration was different between normalglycaemic and prediabetic groups with this being higher in the prediabetic group (76 Vs 54 mIU/L). Furthermore, to examine whether serum zinc concentration was associated with the HOMA parameters, steady state beta cell function (%B), insulin sensitivity (%S) and insulin resistance (IR) for normoglycaemic and pre-diabetic groups were calculated. The pre-diabetic group had a lower median of beta cell function and median insulin sensitivity than the normoglycaemic group and higher median insulin resistance compared to the normoglycemic group.

**Table 2:** Laboratory findings and Homeostasis Model Assessment (HOMA) using for beta cell efficiency in normal and pre-diabetic groups.

	Laboratory findings			Beta cell efficiency using HOMA-2 calculator		
Patient status	Mean fasting blood glucose mmol/l ± SD (Median)	Mean serum zinc, umol/L ±SD (Median)	Mean serum insulin, mIU/L ±SD (Median)	Mean % of beta cell function ±SD (Median)	Mean % of insulin sensitivity ±SD (Median)	Mean insulin resistance ±SD (Median)
**Normal (n = 152)**	4.8±0.4 (4.8)	13.11±1.72 (13)	63.86±39.33 (54)	115.67±46.46 (110.7)	146.33±217.80 (98.5)	1.17±0.71 (1.01)
**Prediabetic (n = 151)**	5.9±0.3 (5.8)	13.15±1.67 (13)	89.95±61.86 (76)	93.55±46.16 (88.8)	141.08±310.30 (68.5)	1.72±1.14 (1.46)
**Diabetic (n = 149)**	7.1±2.1 (6.8)	13.46±3.61 (13)	-	-	-	-
**Non-parametric Kruskal-Wallis P value**	†<0.001	†>0.05	<0.001	<0.001	<0.001	<0.001

Across all groups.

The non-parametric Kruskal-Wallis test was used to test differences by glycaemic status (Table 2). As median serum zinc concentration was similar across all groups no statistical significant difference was found. However, median insulin concentration was significantly different between normoglycaemic and prediabetic groups (p<0.001). Moreover, all the HOMA parameters i.e, beta cell function (%B), insulin sensitivity (%S) and insulin resistance (IR) were found to be statistically significantly different between groups [p<0.001 (table 2)].

Multiple linear regression analyses for HOMA parameters was carried out separately by the participants' glycaemic status i.e normoglycaemic and prediabetics groups (Table 3 & 4). After adjusting for multiple possible confounders, the prediabetic group showed significant association between serum zinc concentration and insulin sensitivity (p = 0.01) indicating that insulin sensitivity increases with an increase in serum zinc concentration. Significant positive association of household income with beta cell function (p = 0.03) and insulin resistance (p = 0.04) was also observed in the prediabetic group. The same analysis also showed that insulin resistance increases with increasing number of medications (p = 0.02). However, similar stratified analysis for the normoglycaemic group did not show any significant association of HOMA parameters with serum zinc or household income.

**Table 3:** Adjusted linear regression analysis for HOMA parameters in participants with normal fasting glucose.

	HOMA2 Parameters								
Exposure indicators	Beta Cell function			Insulin Sensitivity			Insulin Resistance		
	Coefficient	P value	95% CI†	Coefficient	P value	95% CI†	Coefficient	P value	95% CI†
**Serum Zinc**	1.43	0.55	−3.30 to 6.17	9.05	0.35	−9.94 to 28.03	−0.007	0.93	−0.064 to 0.059
**Age**	0.27	0.66	−0.93 to 1.47	−1.82	0.46	−6.62 to 3.00	0.003	0.65	−0.012 to 0.019
**Gender**	4.77	0.59	−12.51 to 22.04	−31.16	0.37	−100.41 to 38.08	−0.056	0.62	−0.28 to 0.17
**BMI**	2.91	0.001	1.27 to 4.56	−2.64	0.43	−9.22 to 3.95	0.050	<0.001	0.028 to 0.071
**Education**	−0.01	0.80	−0.07 to 0.05	0.03	0.84	−0.23 to 0.28	−0.0003	0.40	−0.001 to 0.0005
**House hold income**	−0.83	0.74	−5.72 to 4.05	3.73	0.71	−15.84 to 23.31	−0.007	0.82	−0.070 to 0.056
**Smoking class**	8.55	0.22	−5.12 to 22.22	−80.79	0.004	−135.60 to −25.98	0.22	0.02	0.04 to 0.39
**Alcohol use**	−5.61	0.22	−14.62 to 3.41	29.02	0.11	−7.13 to 65.17	−0.079	0.18	−0.19 to 0.037
**Constant**	−6.98	0.91	−128.90 to 114.92	256.87	0.30	−231.74 to 745.48	−0.398	0.62	−1.975 to 1.178

CI: Confidence Interval.

**Table 4:** Adjusted linear regression analysis for HOMA parameters in pre-diabetic participants.

	HOMA2 Parameters								
Exposure indicators	Beta Cell function			Insulin Sensitivity			Insulin Resistance		
	Coefficient	P value	95% CI†	Coefficient	P value	95% CI†	Coefficient	P value	95% CI†
**Serum Zinc**	0.45	0.85	−4.34 to 5.25	48.63	0.01	10.59 to 86.67	0.135	0.06	−0.007 to 0.28
**Age**	0.83	0.20	−0.445 to 2.10	−4.50	0.36	−14.59 to 5.58	0.011	0.51	−0.022 to 0.043
**Gender**	12.45	0.16	−5.15 to 30.05	−103.95	0.12	−243.50 to 35.60	0.092	0.71	−0.40 to 0.58
**BMI**	2.18	0.009	0.560 to 3.80	−3.39	0.66	−16.27 to 9.48	0.030	0.17	−0.013 to 0.074
**Education**	−3.25	0.34	−9.92 to 3.41	12.23	0.53	−40.63 to 65.09	−0.128	0.18	−0.31 to 0.06
**House hold income**	4.81	0.03	0.50 to 9.12	−12.28	0.46	−46.45 to 21.88	0.126	0.04	0.003 to 0.248
**Smoking class**	3.64	0.57	−9.11 to 16.40	−96.90	0.06	−198.04 to 4.24	−0.017	0.92	−0.357 to 0.322
**Alcohol use**	0.42	0.92	−7.49 to 8.32	−17.34	0.59	−80.04 to 45.36	0.022	0.84	−0.201 to 0.246
**No. of medicatons**	_	_	_	_	_	_	0.114	0.02	0.018 to 0.210
**Constant**	−71.47	0.34	−218.43 to 75.50	168.84	0.77	−996.58 to 1334.25	−2.44	0.23	−6.474 to1.583

CI: Confidence Interval.

We also found that beta cell function was higher in those with higher BMI in both the nomoglycaemic (p = 0.001) and prediabetic (p = 0.009) group. In the normoglycaemic group, insulin resistance (p<0.001) was also higher in those with higher BMI. Moreover, an association was found between smoking status and insulin sensitivity in the normoglycaemic group indicating that current smokers had lower insulin sensitivity (P = 0.004) and that insulin resistance increases with an increase in the number of medications (p = 0.02).

## Discussion

This study has demonstrated that higher serum zinc concentration is associated with increased insulin sensitivity even after adjusting for a number of potential confounders. Furthermore, we also found that the higher the BMI, the higher the beta cell function in both groups and the higher the insulin resistance in the normoglycaemic group. Current smoking status in the normoglycaemic group was associated with lower insulin sensitivity and higher insulin resistance compared to those who had never smoked or were previous smokers. In the prediabetic group, increase in household income led to increase in beta cell function and insulin resistance.

Previous studies have reported reduced concentrations of serum zinc in prediabetics and diabetes mellitus [23], [24] however, like other studies in both developed and developing countries [25], [26], our study did not find any difference in serum zinc concentration amongst the diabetic, pre-diabetic and normoglycaemic groups. Zargar et al found that serum zinc concentration was not altered in diabetes mellitus whereas Ekmekcioglu et al found a higher zinc concentration in whole blood and plasma, although the levels between healthy and diabetic individuals did not differ statistically in these blood fractions. Serum zinc concentration varies under the influence of lifestyle factors such as smoking and alcohol consumption [27], as well as metabolic and hormonal influences [25].

Another method that is broadly used to assess zinc status is the determination of zinc concentration in urine. Hyperzincuria has been described to be a regular finding in type 2 diabetes and has been associated with hyperglycemia [5], [9]. Increased urinary loss can imply a decrease in total body zinc. The serum zinc concentrations in our participants were within normal limits (10–18 umol/L); however as we did not measure zinc excretion, altered levels of zinc in the tissues cannot be ruled out. However, mean GFR was above 70 mls/min in all three groups and the absence of a significant difference in GFR between the groups suggests that there is no impairment in renal function and hence there is unlikely to be a difference in urinary zinc excretion secondary to diabetic nephropathy.

Another explanation for these findings could be the dietary habits of the Australian study population; those involved in this study may all consume food with relatively high levels of zinc. Although zinc deficiency is widespread and 33% of the world's population is affected [28], a recent study on the global prevalence of zinc deficiency showed that in Australia, the estimated prevalence of inadequate zinc intake was <15% [29] whereas in developing countries it was >25%. In developing countries, pulses and cereals represent the major source of zinc. However, in the United States and other developed countries, meat provides 40–60%, pulses about 20–40% and dairy products about 10–30% of the dietary zinc [30]. Although the sample of people in this study was randomly selected, they may have been relatively healthy and hence zinc replete.

We also examined total serum iron concentration amongst the groups, as zinc is known to interact with iron and decrease zinc absorption [31]. The mean total serum iron was 17.47 (±5.38) µmol/L and there was minimal difference in the mean total serum iron concentration between the groups and no statistical difference was seen (p = 0.13, results not presented).

The HOMA parameters findings however indicate that zinc may play a role in progression of diabetes with results showing that serum zinc concentration is associated with insulin sensitivity in the pre-diabetic group. There are many probable mechanisms for this; zinc is important with regard to metabolic diseases such as insulin resistance, metabolic syndrome (MS) and diabetes mainly because it is required for insulin storage in the pancreas and stabilization of insulin hexamers. The anti-oxidative properties of zinc may also delay progression of insulin resistance and diabetes [32]. Furthermore, some studies have suggested that zinc supplementation may improve insulin sensitivity [33], [34] and that zinc status affects some risk factors related to insulin resistance [35].

This study found that increasing BMI was associated with increasing beta cell function in both groups and also an increase in insulin resistance in the normoglycaemic group. Previous studies showed that higher BMI was associated with insulin resistance with or without other associated conditions [36]–[38]. Release of chemokines and inflammatory cytokines from adipose tissue in obesity may result in chronic systemic low grade inflammation that leads to the development of insulin resistance [36]. On the other hand there are still areas of significant uncertainty and gaps of knowledge that limit a full understanding of beta cell function in obesity. It has been found that obesity is associated with a modest expansion of beta cell mass, however any effect of various factors (e.g. duration of obesity or recent changes in body weight) on beta cell mass in humans is unknown [39].

The result of this study also showed that there was an association between current smoking status and insulin sensitivity and insulin resistance. This finding is similar to a recent study which showed that smokers were less insulin sensitive compared with nonsmokers;the mechanisms responsible for this are unclear [40]. Bergman et al. also reported that smoking cessation is associated with an improvement in insulin sensitivity in the absence of changes in adiposity or body weight. Since the association between the number of medications consumed and increased insulin resistance was only observed in the prediabetic group, this is likely an indication of metabolic disorders and associated risks, e.g. obesity, hypertension, lipid abnormalities, and atherosclerotic cardiovascular disease [41].

A strength of this study is the large sample size with a similar number of participants in all groups compared to previous studies. Zinc measurement was done using ICP-MS rather than atomic absorption spectroscopy (AAS) as studies have showed that ICP-MS provides much lower detection limits, high precision, high accuracy and reliable isotopic analysis compared to other methods for measuring trace elements [42], [43]. Also, the coefficients of variation (CV%) for ICP-MS for serum zinc analysis was 13% which indicates low laboratory error. A potential limitation of our study is the absence of information on family history of diabetes and we did not consider other important trace elements such as copper, which may influence serum zinc concentration.

In conclusion, though no significant difference was found in the serum zinc concentration between normoglycaemic, prediabetes, and diabetes groups, this study suggests that zinc may still plays a vital role in diabetes as higher serum zinc concentration is associated with increased insulin sensitivity. The associations of BMI with beta cell function, and insulin sensitivity with smoking status should be explored further as these are important risk factors that may be modified as a means of preventing the progression to full diabetes. Further studies are needed to confirm these findings and the results need to be replicated in longitudinal studies.
